# Explaining reviewing effort: Existing reviews as potential driver

**DOI:** 10.1007/s12525-022-00595-3

**Published:** 2022-10-25

**Authors:** Christoph Rohde, Alexander Kupfer, Steffen Zimmermann

**Affiliations:** 1 Department of Information Systems, Production and Logistics Management, University of Innsbruck, 6020 Innsbruck, Austria; 2 Department of Information Systems, Production and Logistics Management and Digital Science Center (DiSC), University of Innsbruck, 6020 Innsbruck, Austria; 3 Institute of Business Analytics, Ulm University, 89081 Ulm, Germany

**Keywords:** Online reviews, Reviewing effort, Online review platform, Existing reviews, L81, L86

## Abstract

Online review systems try to motivate reviewers to invest effort in writing reviews, as their success crucially depends on the helpfulness of such reviews. Underlying cognitive mechanisms, however, might influence future reviewing effort. Accordingly, in this study, we analyze whether existing reviews matter for future textual reviews. From analyzing a dataset from Google Maps covering 40 sights across Europe with over 37,000 reviews, we find that textual reviewing effort, as measured by the propensity to write an optional textual review and (textual) review length, is negatively related to the number of existing reviews. However, and against our expectations, reviewers do not increase textual reviewing effort if there is a large discrepancy between the existing rating valence and their own rating. We validate our findings using additional review data from Yelp. This work provides important implications for online platforms with review systems, as the presentation of review metrics matters for future textual reviewing effort.

## Introduction

Online consumer reviews strongly influence purchase decisions. Approximately 80% of consumers typically read online reviews before a purchase (Smith & Anderson, 2016), and online reviews are considered as an important information source in online shopping (Rowe & Kingstone, 2018). They are particularly important in online markets which do not allow tangible experiences before consumption; in this context, there are substantial information asymmetries (Hong & Pavlou, 2014). For such markets, the reduction of these information asymmetries by increasing review helpfulness has been shown to impact future sales performance (Yu et al., 2010), and to reduce the costs associated with product returns (Sahoo et al., 2018). However, only a minority of consumers submit reviews (Hu et al., 2009). Even when reviews are written, they are typically short and lack helpful information (Askalidis et al., 2017; Mudambi & Schuff, 2010). Although review system designers would like their reviewers to spend more effort in writing textual reviews as this can be directly related to review helpfulness (Wang et al., 2012), reviewers generally do not invest sufficient effort (Cao et al., 2011).

**Fig. 1:**
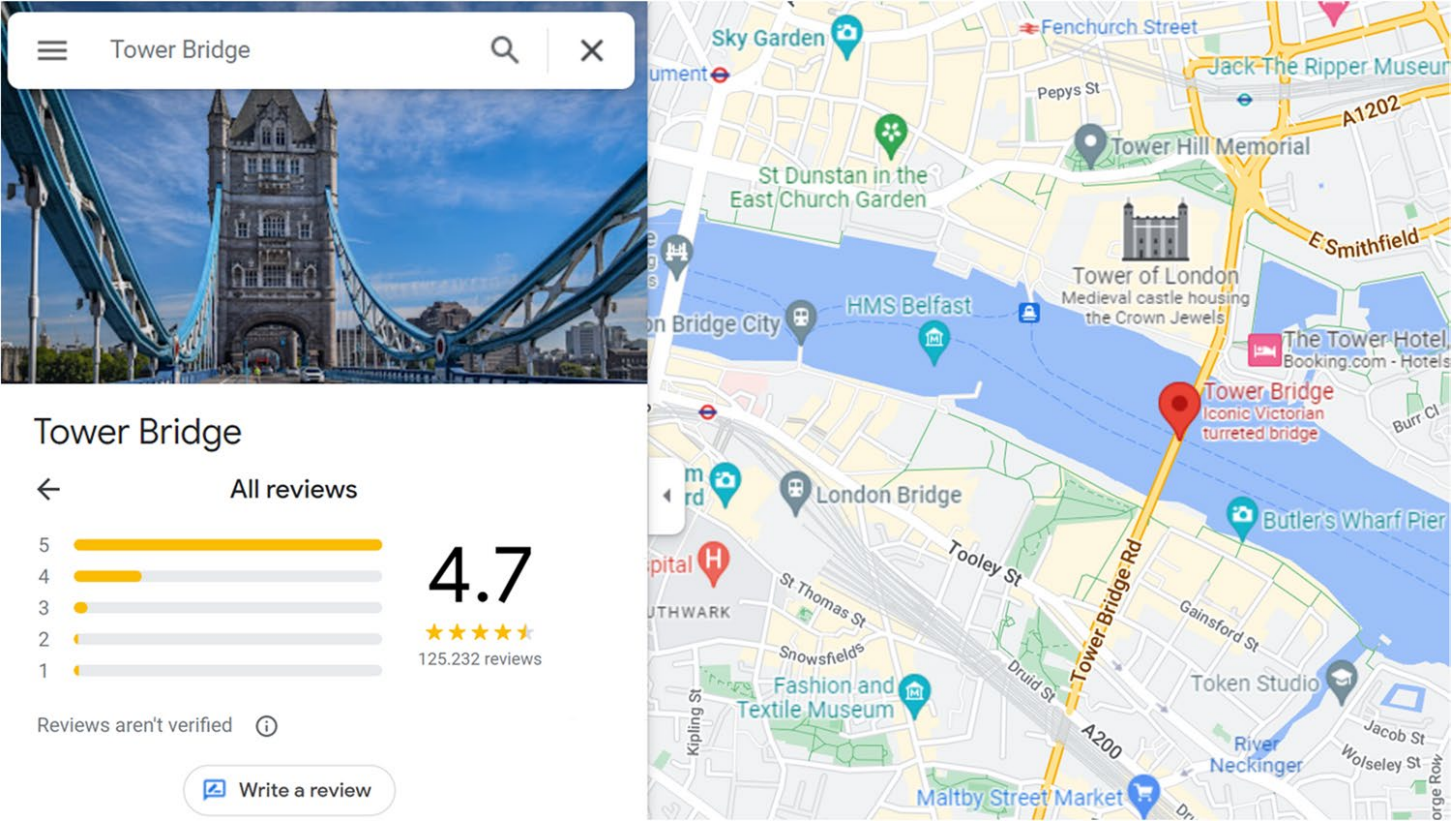
Salience of existing reviews in review systems

Hence, review system designers need to understand what drives the textual reviewing effort of reviewers to ensure the success of their review system. Accordingly, it is important to examine reviewers’ underlying cognitive mechanisms when observing existing reviews before they provide reviews themselves. Figure 1, for instance, shows an example of how reviewers observe the Tower Bridge in London on Google Maps before deciding whether to review.

In this example, when a potential reviewer observes that over 100,000 reviews already exist, why should she invest significant effort into writing a textual review? Similarly, if the vast majority of existing reviews is positive and her experience is also positive, what help is writing another five-star review? We address these questions in the present study. In other words, we focus on situations in which reviewers have already decided to provide ratings, and examine the effort they invest in (textual) reviewing by answering the following research question:

*How do existing reviews influence textual reviewing effort?*

We measure the textual reviewing effort with two variables: whether reviewers decide to write an optional textual review in addition to their star rating and, if they do so, the length of the textual review. Both variables represent important factors for review helpfulness and, consequently, affect the overall success of a review system.

As highlighted in Fig. 1, the number of existing reviews and rating valence are very salient in review systems such as Google Maps or TripAdvisor. Therefore, we expect these two metrics to be the most salient for reviewers’ underlying cognitive mechanisms when observing an object to review. We draw on the collective effort model of Karau and Williams (2001) to hypothesize the effects of the number of existing reviews on the textual reviewing effort. The model describes individual “social loafing” behavior in communities and predicts, transferred to our context, that a high number of existing reviews will decrease reviewing effort. To develop our hypotheses for the rating valence of existing reviews, we apply a combination of expectation disconfirmation theory and balance theory. The former theory describes individuals’ satisfaction when their own experiences and previously generated expectations do not match (Bhattacherjee, 2001; Oliver, 1977). The latter theory argues that individuals experiencing cognitive dissonance want to restore balance (Heider, 1946; Newcomb, 1953). As such, whenever the experience of reviewers is opposite to the rating valence of existing reviews, we expect to observe a higher textual reviewing effort.

We empirically test our hypotheses using online reviews from Google Maps and analyze 37,309 reviews over a period of 12 months. We confirm our hypotheses for the first metric for existing reviews (i.e., number of existing reviews), as we observe a negative association between the number of existing reviews and textual reviewing effort. A high number of existing reviews decreases the probability of a reviewer writing a textual review and, if a textual review is submitted, leads to shorter texts. However, for the second metric (i.e., rating valence of existing reviews), we find no support for our hypotheses, implying that reviewers do not invest more effort if their own experience does not match the expectations formed by the rating valence of existing reviews. To generalize our findings, we repeat our empirical analysis with another review dataset from Yelp, which confirms our main findings. To investigate the potential reasons for the lack of support for our hypotheses on the rating valence of existing reviews, we also exploratively examine the content of reviews where the reviewer’s own experience does not match the expectations from the rating valence of existing reviews in comparison with those where the reviewer’s own experience is more aligned with such expectations. We only sporadically find explicit references to mismatches between expectations and one’s own experience, and we observe that the corresponding reviews are not longer than other reviews. Hence, reviewers do not invest more effort in restoring the balance by outlining arguments, for instance, why other reviews are wrong from the reviewer’s perspective.

This study has two important theoretical implications. First, our findings suggest that the collective effort model is applicable for describing the amount of effort that reviewers invest in the collective task of reviewing. Although Dellarocas et al. (2010) observed that this model is valid for predicting the propensity to review at all in the case of niche products (i.e., typically products with a low number of reviews), we provide evidence that these underlying cognitive mechanisms can also be transferred to textual reviewing effort, and do not stop at the initial decision whether to review or not. Furthermore, Dellarocas et al. (2010) observed a higher propensity to review if products are popular (i.e., typically products with a high number of reviews). This suggests that there existed a U-shaped relationship between the propensity to review and their measure of product popularity, indicating that other cognitive mechanisms must also be present for the initial decision to review. Our results indicate that this is not the case for textual reviewing effort: we do not observe a U-shaped pattern between the decision to write an optional textual review or the actual length of the textual review and the number of existing reviews. Our second theoretical implication relates to expectation disconfirmation theory. The existing literature uses expectation disconfirmation theory to explain whether a reviewer is more likely to submit a positive or negative rating (Ho et al., 2017). Similar to this study, Li et al. (2020) investigated the effects of expectation disconfirmation on the textual review length and found support for the expected underlying mechanism. By using a different, more stringent econometric approach, the results of our study suggest that we cannot apply the expectation disconfirmation theory to explain textual reviewing effort. We find no evidence for the effects of expectation disconfirmation on neither one of the two measures used for the textual reviewing effort, nor for one of the two review datasets used in our analysis.

These findings also have practical implications. For example, review system designers may reconsider the presentation of review metrics to avoid social loafing, e.g., by specifically highlighting the number of existing reviews if the number is low. Similarly, they can introduce an expiration date for reviews to avoid the number of existing reviews reaching an inflationary high level. Moreover, reviewers can be segmented into subgroups (based on age, language, or purpose of purchase) so as to display a (lower) number of existing reviews for a subgroup rather than the (higher) total number of existing reviews.

## Related literature

Prior research on reviewing behavior has mainly focused on the different intrinsic motivations to review. Balasubramanian and Mahajan (2001), for instance, provided a theoretical framework based on social interaction utility, which postulates that reviewers gain utility through reviewing activities. The authors distinguished between the different types of reviewer utilities that could be obtained by writing online reviews. Hennig-Thurau et al. (2004) extended this framework by including two additional utility types and deriving particular motives for each reviewer’s utility type. Although these frameworks are useful in explaining the initial motivations to act as reviewers, they neglect how existing reviews influence the reviewers’ own reviewing behaviors. Studies directly or indirectly addressing the impact of existing reviews are relatively scarce.

For the number of existing reviews, we are only aware of a study by Dellarocas et al. (2010), who examined whether the propensity for reviewers to review at all differs according to product popularity, as this is likely to be correlated with the number of existing reviews. Based on an archival dataset of online movie reviews, the authors found that reviewers are more likely to contribute a review for products that are less available and/or less successful in the market (i.e., niche products), but also for products that are very popular. For the lower-end product (i.e., niche products), the authors used the collective effort model to explain the higher contribution. For the higher-end products (i.e., popular products), they used message involvement theory. The authors ultimately concluded that a U-shaped relationship between the propensity to review at all and product popularity (as measured by box office revenues) exists, thus being indirectly related to the number of existing reviews.

In contrast, Guo and Zhou (2016) and Ho et al. (2017) investigated the effects of the rating valence of existing reviews on actual reviewing behaviors. Ho et al. (2017) used the expectation disconfirmation theory to hypothesize that the disconfirmation experienced (i.e., the discrepancy between the expectation formed by the rating valence of existing reviews and one’s own experience) by a reviewer influences whether to submit a review at all. Their results suggest that reviewers are more likely to review when disconfirmation exists, and that this condition also amplifies the direction of the rating. Related to these studies, Nam et al. (2020) qualitatively surveyed TripAdvisor reviewers and found that expectation disconfirmation is a strong motivation to submit a review (at all) when the disconfirmation is negative (i.e., when the reviewer’s own experience is worse than the expectations generated through the existing rating valence). However, when the disconfirmation is positive, other factors (such as helping others) are more important.

All of these studies highlighted the importance of existing reviews for future review behaviors: whether reviewers submit a review at all (Dellarocas et al., 2010; Ho et al., 2017; Nam et al., 2020), or whether reviewers give a positive or negative rating (Guo & Zhou, 2016; Ho et al., 2017). However, they did not examine whether existing reviews influenced the effort that reviewers spent writing textual reviews. There exists, to the best of our knowledge, only the study by Li et al. (2020) who extend Ho et al.’s (2017) framework and examined textual review length when reviewers’ own experience deviated from the average rating valence of existing reviews. The authors used field data from Yelp as well as a scenario-based experiment, and observed that the discrepancy between one’s own rating and the average rating valence is associated with the length of the respective textual review. Although we acknowledge that Li et al.’s (2020) study captures one aspect of our research (see also Table 1), it is important to highlight that their approach to identifying expectation disconfirmation differs considerably from our approach. We describe the differences in Sect. ‘Contrasting our findings with Li et al.’s (2020) findings’.

**Table 1:** Categorization of related literature and identification of research gaps

	*Reviewing behavior*		*Textual reviewing effort*	
	Propensity to review at all	Actual rating	Write an optional textual review	Textual review length
Number of existing reviews	Dellarocas et al. (2010) (product popularity)		This study	This study
Rating valence of existing reviews	Nam et al. (2020); Ho et al. (2017)	Guo and Zhou (2016) Ho et al. (2017)	This study	Li et al. (2020); This study

Table 1 classifies the related studies, and highlights the research gaps in the literature addressed in this study. Although there is some evidence on the effects of existing reviews on reviewing behavior, there remains a lack of understanding of how textual reviewing effort is affected by existing reviews. Considering the enormous economic relevance of online reviews in general and the textual reviewing effort as an important factor for review helpfulness in particular, this study aims to develop an understanding of how the underlying cognitive mechanisms regarding existing reviews impact the effort invested in textual reviews.^1^

## Theoretical background & hypotheses development

In this study, we aim to examine the effects of existing reviews on textual reviewing effort. We define the textual reviewing effort by (i) the propensity to write an optional textual review and (ii) the textual review length. These variables are appropriate for measuring the textual reviewing effort, as the reviewers in our research environment have the option of simply submitting a star rating. If a reviewer voluntarily decides to write an optional textual review, more effort is required. Writing lengthier reviews also requires more effort from the reviewer, and prior research has already measured reviewing effort based on the length of textual reviews (see, e.g., Burtch et al., 2018).

We build on the collective effort model of Karau and Williams (2001) to develop our hypotheses regarding the effect of the number of existing reviews. Reviewing an object can be seen as a collective task accomplished by many different individuals who provide unique perspectives. The aim of this collective task is to provide a complete and informative picture of an object that cannot be accomplished by an individual alone. Therefore, we expect the collective effort model to fit well with a mechanism for influencing the textual reviewing effort. Importantly, the collective effort model describes the underlying psychological mechanisms leading individuals to invest less effort when working collectively than when working individually. This phenomenon, called “social loafing,” is especially relevant in situations in which individuals feel that their individual effort will not have a major impact on the outcome of the collective task (Karau & Williams, 1993), and/or that the evaluation potential of an individual’s effort will be diminished in the collective task (Harkins, 1987).

Applying this model to online reviews, it means that reviewers might feel that their own review has less impact on the overall evaluation of the reviewed object if the number of existing reviews is high. In other words, even though reviewers can see their name linked to their review, they might feel that their individual effort will not have a major impact on the outcome of the collective task if many reviews already exist. Therefore, we expect that the number of existing reviews reduces the reviewers’ own textual reviewing effort. As outlined above, reviewers can submit a star rating in our research environment and, optionally, write a textual review corresponding to their star rating. As writing an additional textual review implies an additional reviewing effort, we formulate Hypothesis 1 as follows.

- **H1**: A higher number of existing reviews decreases the propensity to write textual reviews.

In the same vein, we further hypothesize that this psychological mechanism also influences the amount of effort invested when a textual review is submitted. Although reviewers generally have the option to freely choose the length of their textual reviews, prior research has argued that lengthier reviews require more effort (Burtch et al., 2018). Thus, additionally measuring the review length is important for determining how much effort reviewers invest in a textual review once they have decided to write it. Using the analogous explanation as above, a reviewer may decrease his/her effort for writing a textual review if there is already a high number of existing reviews. In view of this, Hypothesis 2 is formulated as follows.

- **H2**: A higher number of existing reviews decreases the length of a textual review.

Further, we expect that not only is the number of existing reviews a relevant review metric for the textual reviewing effort, but also the rating valence of existing reviews. Reviewers typically develop expectations regarding an object to review based on the rating valence of existing reviews. Thus, an interaction exists between the observed rating valence of existing reviews and the reviewers’ own experiences.

This interaction can be described using expectation disconfirmation theory (Bhattacherjee, 2001; Oliver, 1977). Expectation disconfirmation theory explains the satisfaction of individuals after experiencing and evaluating an object as a function of the disconfirmation of previously generated expectations. Expectation disconfirmation theory states that individuals are more likely to experience a high level of dissatisfaction when the disconfirmation (i.e., the discrepancy between their expectations and their own evaluation of the object) is negative. Positive disconfirmation, on the other hand, leads to a high level of satisfaction (Oliver, 1980). We expect that both positive and negative disconfirmation to be relevant for textual reviewing effort: For this, we draw on balance theory, which states that individuals try to restore balance by resolving cognitive dissonance when holding two conflicting ideas in their minds (Heider, 1946; Newcomb, 1953). In other words, individuals who experience a disconfirmation want to resolve cognitive dissonance and restore balance.

In our context, disconfirmation can arise if reviewers have generated negative (positive) expectations from the existing reviews but then experience a positive (negative) own evaluation. Hence, reviewers hold two conflicting evaluations in their minds. To resolve this cognitive dissonance between the conflicting evaluations of the other reviewers and their own evaluations, individuals can use reviews to restore balance (Hennig-Thurau et al., 2004). While this includes a star rating that corresponds to their own experience (Guo & Zhou, 2016; Ho et al., 2017), reviewers can additionally write textual reviews to further strengthen their attempt to restore balance. In essence, reviewers try to resolve cognitive dissonance and correct opinions in existing reviews by investing more effort into their reviews to communicate regarding the difference between their own evaluation and the evaluation of other reviewers. Thus, we expect that reviewers who experience high disconfirmation are more likely to write additional textual reviews. Therefore, Hypothesis 3 is as follows.

- **H3**: Disconfirmation increases the propensity to write a textual review.

Analogous to the arguments above, we expect that reviewers who experience high disconfirmation and decide to write a textual review will also invest more effort into writing the review itself. When reviewers experience disconfirmation, they might, for instance, provide arguments that explain why the evaluation from existing reviews is incorrect or justify their own evaluation. In sum, we expect that they will invest more effort into writing the review to resolve the cognitive dissonance of the conflicting evaluations and restore balance. Thus, we formulate Hypothesis 4 as follows.

- **H4**: Disconfirmation increases the length of textual reviews.

## Empirical analysis

### Research environment

The Google Maps review system represents a well-suited research environment for our empirical analysis, as textual reviews are not mandatory (i.e., a star rating is sufficient). This allows us to examine two aspects of the reviewing effort: the propensity to write an optional textual review (i.e., Hypotheses 1 and 3) and the length of textual reviews (i.e., Hypotheses 2 and 4). On Google Maps, reviewers can review locations ranging from restaurants and hotels to shops to sights. As restaurants, hotels, and shops are subject to personal taste and depend on individual experiences (e.g., noisy rooms, unfriendly staff), we focused on tourist sights such as bridges or fountains that are relatively less sensitive to time variability than restaurants (e.g., a new chef) or hotels (e.g., renovated rooms). Therefore, we selected 10 bridges, 10 squares, 10 fountains, and 10 monuments across Europe as the relevant locations. We verified that these sights did not charge visitors, were accessible to the public, and were reviewed on Google Maps. We extracted data from Google Maps by scraping all existing reviews for each site. The selected sights were located in 27 different cities across Europe (the full list of sights is outlined in the Appendix).

Unfortunately, reviews on Google Maps do not include a timestamp, but rather relative date information (e.g., one week ago), thereby restricting our period of analysis. More specifically, for all reviews written in the previous year, Google Maps provides monthly relative dates (e.g., 11 months ago). For all reviews older than one year, only yearly relative dates are provided (e.g., two years ago). Therefore, we focused on a period with monthly reviews for our analysis. As the review data were downloaded at the end of November 2017, our relevant period of analysis ranged from December 2016 to November 2017. For each review, we retrieved the review date, star rating, textual review (if available), number of reviews the reviewer had already written, and whether a photo was attached to the review.

### Data preparation and variables

To empirically test our hypotheses, the existing reviews needed to be appropriately aggregated. As noted above, we obtained monthly review data. Therefore, we aggregated the existing reviews on a monthly basis as follows. When starting in the first month with our analysis, we calculated the relevant review metrics that are salient in review systems (see below) using all existing reviews until this month. For the second month, the relevant review metrics are calculated based on all existing reviews until the second month, and so on. Table 2 summarizes the data structure.

**Table 2:** Abstracted data structure

Individual review-specific data							Existing review data		
Review ID	Month	$Rating$	$TextRev$	$RevLength$	$RevExperience$	$Picture$	$NumReviews$	$Imbalance$	$AvgRating$
1	12/2016	4	Yes	150	25	Yes	200	0.50	3.65
2	12/2016	5	No	–	150	Yes	200	0.50	3.65
3	01/2017	3	No	–	3	No	202	0.51	3.67
4	01/2017	5	Yes	100	55	No	202	0.51	3.67
5	01/2017	4	Yes	120	35	Yes	202	0.51	3.67

The first seven columns of Table 2 indicate individual review-specific information, such as the rating or whether a textual review was written. The information on whether a textual review was written ($TextRev$) and textual review length ($RevLength$), measured by the number of characters, represent our dependent variables. From the individual review-specific information, we additionally obtain the individual rating ($Rating$) and reviewing experience – the number of reviews the reviewer had written so far – ($RevExperience$) as control variables. Both are important covariates for the textual reviewing effort, as low-valence reviews are typically longer than high-valence reviews (e.g., Chua & Banerjee, 2015; Ghasemaghaei et al., 2018 or Salehan & Kim, 2016), and reviewers with high reviewing experience write more helpful reviews (e.g., Baek et al., 2012 or Ghose & Ipeirotis, 2011) and longer reviews (Hong et al., 2017). Finally, we also collected information on whether reviewers submitted a picture with their rating ($Picture$). Although the latter information is not used in the main analysis, we will use it as a robustness check in Sect. ‘Robustness’.

The last three columns of Table 2 are the independent variables and represent the review metrics that provide information regarding the existing reviews saliently in online review systems (see also Fig. 1). As outlined above, we recalculate these variables each month to consider the newly emerged reviews. To examine Hypotheses 1 and 2, we use the number of existing reviews ($NumReviews$) as a straightforward measure, as this data is clearly shown in Google Maps. To investigate the effects of expectation disconfirmation (i.e., Hypotheses 3 and 4), we needed to capture the rating valence of existing reviews. The rating valence on Google Maps is represented by the average rating valence and a histogram showing the frequencies of the respective star ratings. As the latter also provides information (at least visually) about the other moments of the rating distribution, we aim to quantify this aspect in our measure of the rating valence as well. For this purpose, we use the imbalance score ($Imbalance$) proposed by Schoenmueller et al. (2020), calculated as the ratio between the number of good ratings (i.e., four-star and five-star ratings) and total number of ratings. In this way, it also serves as “a measure of the skewness of the distribution to the positive side of the scale such that an imbalance measure above 50% means that there are more positive reviews and below 50% indicates a majority of negative reviews.” (Schoenmueller et al., 2020, p. 858). For robustness, we also use the average rating valence ($AvgRating$) as a measure of the rating valence of the existing reviews (see Sect. ‘Robustness’).

Table 3 presents the summary statistics for our data and Table 4 the correlation matrix, respectively.

**Table 3:** Summary statistics

	Observations	Minimum	Maximum	Mean	Median	Std. Deviation
$TextRev$	37,309	0	1	0.46	0	0.50
$RevLength$	17,323	1	319	62	43	56
$NumReviews$	37,309	47	2,738	1,057	919	616
$Imbalance$	37,309	0.78	1	0.88	0.87	0.04
$Rating$	37,309	1	5	4.44	5	0.81
$RevExperience$	37,309	1	6,294	63	19	157
$AvgRating$	37,309	4.13	4.77	4.43	4.44	0.13
$Picture$	37,309	0	1	0.02	0	0.12

This table shows summary statistics for all variables used in the analysis. As textual reviews are not mandatory, the number of observations for textual review length is lower than for the other variables. The first two rows represent the dependent variables. Rows three to six represent the independent variables, including controls. The last two rows represent variables used for robustness checks in Sect. ‘Robustness’

**Table 4:** Correlation matrix

	$\mathrm{TextRev}$	$\mathrm{RevLength}$	$\mathrm{NumReviews}$	$\mathrm{Imbalance}$	$\mathrm{Rating}$	$\mathrm{RevExperience}$	$\mathrm{AvgRating}$
$NumReviews$	-0.10***	-0.04***					
$Imbalance$	0.07***	0.02***	-0.22***				
$Rating$	0.04***	-0.08***	-0.03***	0.13***			
$RevExperience$	0.26***	0.08***	-0.09***	0.04***	-0.02***		
$AvgRating$	0.07***	0.01*	-0.18***	0.96***	0.13***	0.04***	
$Picture$	0.06***	0.06***	-0.06***	0.05***	0.03***	0.07***	0.05***

This table shows the correlation matrix for all variables used in the analysis. Correlations between textual review length and the other variables are based on the subsample of reviews that include a textual review (n = 17,323). All other correlations are based on the full sample (n = 37,309). ^***^ p < 0.01, ^*^ p < 0.1

### Model specification

As outlined above, our dependent variables (i.e., proxies for the textual reviewing effort) are given by the individual reviews and represent whether an optional textual review was written (or not) and the length of the textual review. The main independent variable for Hypotheses 1 and 2 is the number of existing reviews.

To examine whether the number of existing reviews determines the binary decision to write a textual review, we use a logistic regression model and estimate:

1 $${TextRev}_{i,t,j}=\alpha +{\beta }_{1}\mathrm{ln}({NumReviews}_{i,t-1})+{\beta }_{2}{Rating}_{i,t,j}+{\beta }_{3}\mathrm{ln}\left({RevExperience}_{i,t,j}\right)+{\upgamma }_{\mathrm{t}}+{\epsilon }_{i,t,j},$$

where ${TextRev}_{i,t,j}$ represents a binary variable being 1 if review $j$ for sight $i$ in month $t$ includes a textual review, and 0 otherwise. $\mathrm{ln}({NumReviews}_{i,t-1})$ represents the natural logarithm of the total number of existing reviews before month $t$ (i.e., month $t-1$) for sight $i$. As control variables, we use ${Rating}_{i,t,j}$ as an indicator variable which depicts the individual ratings for review $j$, and $\mathrm{ln}({RevExperience}_{i,t,j})$ which is the natural logarithm of the number of reviews the reviewer has already written. The log-transformation is applied because both total number of existing reviews and reviewing experience are (strongly) positively skewed. ${\upgamma }_{\mathrm{t}}$ represents month dummies to account for potential seasonal effects^2^ and ${\epsilon }_{i,t,j}$ describes the remaining error term. For this estimation and all subsequent estimations, we use robust standard errors clustered at the sight level.

For Hypothesis 2, we use the textual review length as the dependent variable. The number of written characters in a review represents a count variable that can only take positive integer values; therefore, a Poisson regression model would be an appropriate estimator. However, because our data is overdispersed, a negative binomial distribution is preferred to a Poisson distribution. This is because the negative binomial distribution has an additional parameter, also referred to as the negative binomial dispersion parameter (Hilbe, 2014). To additionally account for the fact that there are no “zeros” in the number of written characters (as this implies no textual review at all), we ultimately apply a zero-truncated negative binomial regression model. In principle, a zero-truncated negative binomial distribution is represented by a negative binomial distribution for which the probability of a zero count is subtracted (Hilbe, 2014). Except for the other proxy for textual reviewing effort (i.e., textual review length) as the dependent variable and the other estimation model, all remaining variables and specifications for testing Hypothesis 2 are the same as those for Eq. (1).

Although we use the same dependent variables for Hypotheses 3 and 4, the effect of the expectation disconfirmation cannot be directly measured based on one independent variable. Specifically, it requires a combination of (i) a reviewer having a positive or negative experience and (ii) the rating valence of existing reviews being opposed to the reviewer’s experience. As outlined above, we use the imbalance score to measure the existing rating valence, and we obtain the reviewer’s experience through his/her individual rating. In other words, we are interested in the coefficient of the interaction between one’s own experience (as measured by the individual rating) and the rating valence of existing ratings (as measured by the imbalance score). Importantly, we still need to control for the individual rating as well, as it represents a predictor for the reviewing effort per se, independent of whether a disconfirmation was experienced (see, e.g., Salehan & Kim, 2016).

For Hypothesis 3, we therefore extend the logistic regression model outlined in Eq. (1) as follows:

2 $$\begin{aligned}{TextRev}_{i,t,j}=& \alpha +{\beta }_{1}\mathrm{ln}({NumReviews}_{i,t-1})+{\beta }_{2}{Rating}_{i,t,j}\times {Imbalance}_{i,t-1}+{\beta }_{3}{Rating}_{i,t,j}\\ &+ {Imbalance}_{i,t-1}+{\beta }_{4}\mathrm{ln}\left({RevExperience}_{i,t,j}\right)+{\upgamma }_{\mathrm{t}}+{\epsilon }_{i,t,j}\end{aligned}$$

where, in addition to the variables in Eq. (1), ${Imbalance}_{i,t-1}$ and ${Rating}_{i,t,j}\times {Imbalance}_{i,t-1}$ is added. The interaction between the specific individual rating for review $j$ for sight $i$ in month $t$ and ${Imbalance}_{i,t-1}$ (measuring the rating valence of the existing ratings in month $t-1$ for the same sight $i$) captures the effect of expectation disconfirmation on the propensity to write an optional textual review. To avoid multicollinearity issues, we mean-center the imbalance score.

For Hypothesis 4, we again apply the zero-truncated negative binomial model with the textual review length as the dependent variable. However, we also include ${Imbalance}_{i,t-1}$ and the interaction term ${Rating}_{i,t,j}\times {Imbalance}_{i,t-1}$ to measure the effect of the expectation disconfirmation on textual review length.

### Results

Table 5 presents the main results from the analysis. The statistically significant coefficient in Column (i) for $\mathrm{ln}(NumReviews)$ indicates that an increase in the number of reviews is negatively related to the propensity to write an optional textual review. This confirms Hypothesis 1, i.e., that the number of existing reviews is negatively associated with reviewers’ propensity to write an optional textual review. Regarding the control variables, we observe that reviewing experience is positively related to the propensity to write an optional textual review. Although this is in line with Hong et al.’s (2017) finding that reviewers with high reviewing experience also write longer reviews, it could also simply capture reviewers’ tendency to write textual reviews. Furthermore, existing literature documents that low-valence reviews are typically longer than high-valence reviews (e.g., Chua & Banerjee, 2015; Ghasemaghaei et al., 2018 or Salehan & Kim, 2016). Interestingly, we observe a different pattern for the propensity to write an optional textual review: An individual rating that is both either very positive (i.e., five-star ratings) or very negative (i.e., one-star ratings and two-star ratings) is positively related to the propensity to write an optional textual review.

**Table 5:** Main results

	(i)	(ii)	(iii)	(iv)
Dependent variable:	$TextRev$	$RevLength$	$TextRev$	$RevLength$
$\mathrm{ln}(NumReviews)$	-0.223***	-0.063**	-0.188***	-0.052*
	(0.056)	(0.027)	(0.060)	(0.025)
$Imbalance$			-1.770*	-0.827
			(1.045)	(0.589)
$Ratin{g}_{1 Star}\times Imbalance$			-2.305	3.697**
			(3.971)	(1.553)
$Ratin{g}_{2 Stars}\times Imbalance$			5.304**	-0.214
			(2.246)	(1.131)
$Ratin{g}_{3 Stars}\times Imbalance$			(omitted)	(omitted)
$Ratin{g}_{4 Stars}\times Imbalance$			2.987***	0.350
			(1.035)	(0.522)
$Ratin{g}_{5 Stars}\times Imbalance$			4.286***	1.881***
			(1.033)	(0.492)
$\mathrm{ln}(RevExperience)$	0.802***	0.060***	0.800***	0.059***
	(0.018)	(0.006)	(0.019)	(0.006)
$Ratin{g}_{1 Star}$	0.865***	0.113	0.839***	0.155*
	(0.136)	(0.085)	(0.143)	(0.089)
$Ratin{g}_{2 Stars}$	0.540***	0.153**	0.624***	0.145**
	(0.129)	(0.065)	(0.139)	(0.069)
$Ratin{g}_{3 Stars}$	(omitted)	(omitted)	(omitted)	(omitted)
$Ratin{g}_{4 Stars}$	0.067	-0.033	0.091*	-0.022
	(0.061)	(0.024)	(0.054)	(0.025)
$Ratin{g}_{5 Stars}$	0.437***	-0.150***	0.449***	-0.148***
	(0.065)	(0.032)	(0.052)	(0.028)
Constant	-1.575***	4.359***	-1.802***	4.285***
	(0.346)	(0.167)	(0.364)	(0.151)
Pseudo-R-squared	0.212	0.002	0.213	0.002
Observations	37,309	17,323	37,309	17,323
Model	LOGIT	ZTNB	LOGIT	ZTNB

Three-star ratings are used as base for the individual rating (Columns (i)–(iv)) and the interaction terms (Columns (iii)–(iv)) and are therefore omitted. Columns (i) and (iii) represent a logistic regression model (LOGIT). Columns (ii) and (iv) represent a zero-truncated negative binomial regression model (ZTNB). Pseudo-R-squared represents McFadden’s pseudo-R-squared. Robust standard errors clustered at the sight level in parentheses. ^***^ p < 0.01, ^**^ p < 0.05, ^*^ p < *0.1*

For the textual review length, we also observe a statistically significant coefficient in Column (ii) for $\mathrm{ln}(NumReviews)$. This finding suggests that the number of reviews is also negatively related to the textual review length, confirming Hypothesis 2. The coefficient for reviewing experience is again positive and statistically significant. For the individual rating, we observe a different picture than before, as very positive ratings (i.e., five-star ratings) are negatively related to the textual review length. In contrast, two-star ratings are positively related to textual review length. The insignificant coefficient of the one-star ratings might be related to the low number of 1-star ratings submitted in our dataset (only 1.4% of all ratings are one-star ratings). To account for this issue, we generate a less granular rating variable ranging from 1 to 3, where “1” includes one- and two-star ratings, “2” includes all three-star ratings, and “3” includes four- and five-star ratings. While the results for the number of reviews do not change, the less granular rating variable exhibits the expected coefficients: rating category “1” is significantly positively related and rating category “3” is significantly negatively related to textual review length (results not tabulated).

**Fig. 2:**
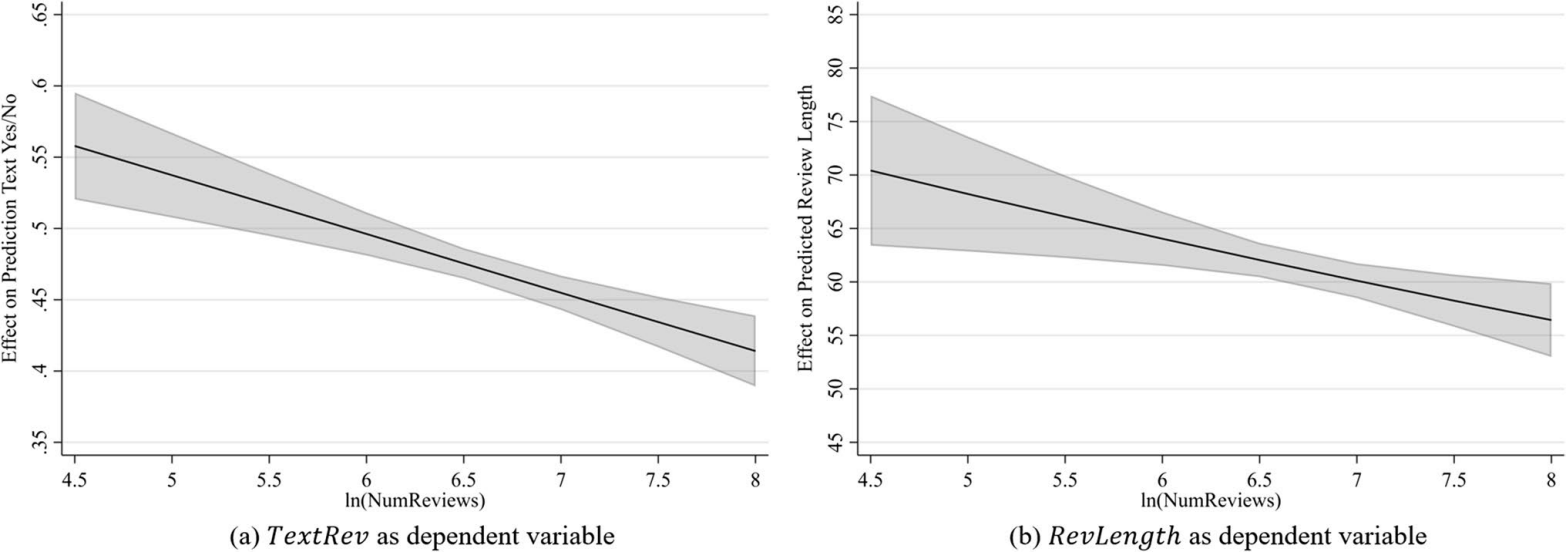
Average marginal effects

Figure 2 shows the marginal effects of the number of reviews on the propensity to write an optional textual review (Panel a) and on the textual review length (Panel b). The figure illustrates the effect of the negative coefficients for the number of existing reviews: the higher the number of existing reviews, the lower the textual reviewing effort. Hence, for a sight that has approximately 90 ($=\mathrm{exp}(4.5)$) existing reviews, the expected propensity to write an optional textual review is, c. p., 56% and the expected textual review length is, c. p., 70 characters. In contrast, for a sight with approximately 2980 ($=\mathrm{exp}(8)$) existing reviews, the expected propensity to write an optional textual review decreases to, c. p., 42%, and the expected textual review length decreases to, c. p., 57 characters.

Column (iii) of Table 5 presents the main results for Hypothesis 3. The coefficients of interest are the imbalance score and its interaction with the star ratings. Since we use three-star ratings as base for the individual ratings, the main effect of the imbalance score also captures the interaction with three-star ratings. Although this coefficient is barely significant ($p=0.09$), we would expect to observe the effect of disconfirmation at both extreme ends of the star ratings. To be specific, we would expect the coefficient for $Ratin{g}_{1 Star}\times Imbalance$ and $Ratin{g}_{2 Stars}\times Imbalance$ to exhibit a positive coefficient, because a bad experience in combination with a high imbalance score (i.e., the existing rating valence is very positive) should be associated with high textual reviewing effort. For the coefficients of $Ratin{g}_{4 Stars}\times Imbalance$ and $Ratin{g}_{5 Stars}\times Imbalance$, we would expect them to be negative, because a good experience in combination with a high imbalance score should be associated with low textual reviewing effort (as it confirms the expectation generated from existing reviews). However, the results in Column (iii) do not support our hypothesis. The coefficients of the interaction terms are either not significant (one-star ratings), or are significant but do not exhibit the expected sign (four- and five-star ratings). Importantly, the coefficients for number of existing reviews and for all control variables exhibit significant levels, similar to those in Column (i).

Finally, Column (iv) of Table 5 presents the results for Hypothesis 4, with the textual review length as the dependent variable. Based on our theory, we would again expect the same pattern for the interaction terms as in Hypothesis 3. While we observe a statistically significant coefficient with the expected sign for the interaction term with one-star ratings, the interaction term with five-star ratings shows a statistically significant coefficient with the same unexpected sign as before.

Hence, there is only very limited evidence for an increase in textual reviewing effort in case of a negative disconfirmation (i.e., for two-star ratings in Column (iii) and one-star ratings in Column (iv)) and a contradictory observation for positive disconfirmation (i.e., for four-star ratings in Column (iii) and five-star ratings in Columns (iii) and (iv)). To better understand the effect sizes, we now compare one-star ratings whose existing reviews exhibit a very low imbalance score (below the first quartile) with the one-star ratings whose existing reviews exhibit a very high imbalance score (above the third quartile). As before, the latter represents the case of negative disconfirmation for which we hypothesize higher textual reviewing effort. One-star ratings with negative disconfirmation exhibit 44.2% textual reviews with an average length of 83 characters. One-star ratings whose existing reviews show a very low imbalance score (i.e., no or less negative disconfirmation) exhibit 42.5% textual reviews with an average length of 57 characters. Although the remarkable difference in average textual review length can also be observed in the coefficient for $Ratin{g}_{1 Star}\times Imbalance$ in Table 5 Column (iv), we caution that there are in total only few one-star ratings in the sample (n = 394) and that the results should not be overinterpreted. When repeating the same exercise for five-star ratings, we observe that five-star ratings with positive disconfirmation (i.e., imbalance score below the first quartile) exhibit 43.3% textual reviews with an average length of 55 characters. For five-star ratings with no or less positive disconfirmation (i.e., imbalance score above the third quartile), we observe 55.2% textual reviews with an average length of 62 characters. These differences help to understand the significant coefficients for $Ratin{g}_{5 Stars}\times Imbalance$ in Table 5 Column (iii) and Column (iv). Given that in sum only two coefficients in Columns (iii) and (iv) show the expected sign for negative disconfirmation and that we consistently observe contradictory coefficients for positive disconfirmation, we conclude that there is no support for Hypotheses 3 and 4.

### Robustness

In this section, we consider alternative specifications to verify the robustness of the results. Note that for the sake of brevity, we only provide the full regression output from testing all hypotheses simultaneously, and do not explicitly show the results for Hypotheses 1 and 2 alone (i.e., Columns (i)–(ii) in Table 5).

First, as outlined in Sect. ‘Data preparation and variables’, the imbalance score captures the rating valence of the existing reviews. Alternatively, one could also simply use the average rating valence, which is often clearly shown in review systems (but neglects other moments of the rating valence distribution). To test this alternative measure, we replace the imbalance score with the average rating ($AvgRating$). As for the imbalance score above, we also mean-center the average rating. The results from this exercise are shown in Columns (i) and (ii) of Table 6. As both a high imbalance score and a high average rating valence indicate that the rating valence of existing reviews is positive, we expect the coefficients for the interaction terms to exhibit the same signs. While we observe one coefficient of the interaction terms (i.e., $Ratin{g}_{2 Stars}\times AvgRating$) to be significant with the expected sign in Column (i), the other coefficients are either not significant, or exhibit a sign that is contrary to our expectations. Similarly, for the textual review length shown in Column (ii), only the coefficient for the interaction term with five-star ratings is statistically significant; however, it again exhibits a positive sign, despite our expectation of a negative sign.

**Table 6:** Robustness tests

	(i)	(ii)	(iii)	(iv)
Dependent variable:	$TextRev$	$RevLength$	$Picture$	$RevLengthZeros$
$\mathrm{ln}(NumReviews)$	-0.185***	-0.055**	-0.707***	-0.178***
	(0.058)	(0.025)	(0.090)	(0.053)
$Imbalance$			6.580	-2.225**
			(4.425)	(0.920)
$Ratin{g}_{1 Star}\times Imbalance$			-25.070***	3.078
			(7.687)	(2.533)
$Ratin{g}_{2 Stars}\times Imbalance$			-18.260***	1.091
			(7.033)	(2.220)
$Ratin{g}_{3 Stars}\times Imbalance$			(omitted)	(omitted)
$Ratin{g}_{4 Stars}\times Imbalance$			-3.565	2.112**
			(4.469)	(0.846)
$Ratin{g}_{5 Stars}\times Imbalance$			-2.303	4.262***
			(4.382)	(1.045)
$AvgRating$	-0.311	-0.242		
	(0.393)	(0.201)		
$Ratin{g}_{1 Star}\times AvgRating$	-0.527	0.830		
	(1.218)	(0.518)		
$Ratin{g}_{2 Stars}\times AvgRating$	1.989**	-0.217		
	(0.806)	(0.372)		
$Ratin{g}_{3 Stars}\times AvgRating$	(omitted)	(omitted)		
$Ratin{g}_{4 Stars}\times AvgRating$	0.737**	0.054		
	(0.360)	(0.165)		
$Ratin{g}_{5 Stars}\times AvgRating$	1.169***	0.527***		
	(0.419)	(0.175)		
$\mathrm{ln}(RevExperience)$	0.801***	0.060***	0.307***	0.475***
	(0.019)	(0.006)	(0.029)	(0.018)
$NumPictures$			0.037***	
			(0.010)	
$Ratin{g}_{1 Star}$	0.852***	0.140	-0.277	0.697***
	(0.148)	(0.093)	(0.609)	(0.098)
$Ratin{g}_{2 Stars}$	0.648**	0.138**	0.249	0.542***
	(0.132)	(0.069)	(0.481)	(0.096)
$Ratin{g}_{3 Stars}$	(omitted)	(omitted)	(omitted)	(omitted)
$Ratin{g}_{4 Stars}$	0.085	-0.024	0.284*	0.026
	(0.056)	(0.024)	(0.164)	(0.047)
$Ratin{g}_{5 Stars}$	0.440***	-0.148***	0.626***	0.098*
	(0.053)	(0.028)	(0.181)	(0.053)
Constant	-1.803***	4.304***	-1.475***	2.662***
	(0.347)	(0.156)	(0.542)	(0.316)
Pseudo-R-squared	0.213	0.002	0.062	0.011
Observations	37,309	17,323	37,309	37,309
Model	LOGIT	ZTNB	LOGIT	NB

Three-star ratings are used as base for the individual rating and the interaction terms and are therefore omitted. Column (i) represents the logistic regression model (LOGIT) with the average rating as measure for rating valence. Column (ii) represents the zero-truncated negative binomial regression model (ZTNB) with the average rating as measure for rating valence. Column (iii) re-estimates the logistic regression model (LOGIT) in Equation (2) with the binary choice of submitting a picture as dependent variable. Column (iv) estimates a negative binomial regression model (NB) and treats reviews without an additional textual review as a textual review with a length of zero. Pseudo-R-squared represents McFadden’s pseudo-R-squared. Robust standard errors clustered at the sight level in parentheses. ^***^ p<0.01, ^**^ p<0.05, ^*^ p<*0.1*

Second, we also obtained information on whether a reviewer submitted a picture with their rating. While we expect that writing a textual review is more effortful, adding a picture still represents some type of additional effort that reviewers are willing to invest. To examine our hypotheses with this type of reviewing effort, we re-estimate Eq. (2) with the binary choice of submitting a picture ($Picture$) as a dependent variable. Because our dataset allows us to sum up the number of existing pictures per sight, we also included this variable ($NumPictures$) in our set of independent variables. The results are shown in Column (iii) of Table 6. We observe that the number of existing reviews is negatively related to the propensity to submit a picture. This provides further evidence for Hypotheses 1 and 2 regarding social loafing, i.e., when there are already many existing reviews, reviewers are less likely to invest more effort by contributing an optional picture. Notably, we also observe that the number of existing pictures is positively related to the propensity to submit an optional picture. Although this actually contradicts our hypothesis of social loafing, it could also be the case that an alternative mechanism is present: it might simply be an indicator of whether the tourist sight is worth taking a picture.

Third, we run separate analyses for the propensity to review and textual review length, respectively. An alternative specification would be to treat those reviews without an additional textual review as a textual review with a length of zero ($RevLengthZeros$). In this vein, we can estimate a negative binomial regression model using the full sample with 37,309 observations.^3^ Column (iv) of Table 6 shows the results for this exercise. First, the alternative regression model confirms our finding regarding the effect of the number of existing reviews on textual reviewing effort. Second, there is again no support for the hypothesized effect of the rating valence of existing reviews on textual reviewing effort as we also observe contradictory signs for both $Ratin{g}_{4 Stars}\times Imbalance$ and $Ratin{g}_{5 Stars}\times Imbalance$.

### Cross-validation with another review dataset

As all of our results are based on a single dataset, one might wonder about the generalizability of our findings. Accordingly, we used another publicly available dataset from Yelp, and repeated the analyses. In particular, we used the most recent dataset from the Yelp Challenge (2021). As for the Google Maps dataset, we needed to prepare the dataset accordingly, and proceeded as follows. Because the Yelp dataset did not include sights, we focused on restaurants only to obtain a homogenous set of reviews. We then selected a time period of two years (i.e., 2018 to 2019). The dataset also included reviews from 2020; however, we did not use them, as they might have been biased owing to the Covid-19 pandemic. For example, reviewers could explicitly write a textual review to highlight the hygienic measures of a restaurant or to describe their (newly established) takeaway service. We aggregated the review data on a monthly basis (analogous to the previous analysis) and required that at least two reviews had been written for the restaurant in each month. Ultimately, we obtained 373,205 reviews for 2,213 restaurants from January 2018 to December 2019. We then prepared the dataset in the same way as described in Sect. ‘Data preparation and variables’, by considering the number of all existing reviews as well as the respective metrics for the rating valence of existing reviews. As writing a textual review is mandatory for Yelp, we could only examine Hypotheses 2 and 4.

The results from this exercise are presented in Table 7. As for the previous section, we jointly examine Hypotheses 2 and 4 by re-estimating the zero-truncated negative binomial regression model, which includes all relevant independent variables. Column (i) presents the results for the main model. First, as for the Google Maps data, we observe that the number of textual reviews is negatively related to textual review length. This finding strongly supports Hypothesis 2. Second, we also find no support for Hypothesis 4 in the Yelp dataset, as none of the interaction terms are statistically significant. Third, the control variables exhibit the same pattern as with the Google Maps data.

**Table 7:** Results for Hypothesis 2 and Hypothesis 4 with Yelp data

	(i)	(ii)
Dependent variable:	$RevLength$	$RevLength$
$\mathrm{ln}(NumReviews)$	-0.054***	-0.054***
	(0.005)	(0.005)
$Imbalance$	0.077	
	(0.070)	
$Ratin{g}_{1 Star}\times Imbalance$	-0.004	
	(0.075)	
$Ratin{g}_{2 Stars}\times Imbalance$	-0.012	
	(0.063)	
$$Ratin{g}_{3 Stars}\times Imbalance$$	(omitted)	
$Ratin{g}_{4 Stars}\times Imbalance$	0.053	
	(0.056)	
$Ratin{g}_{5 Stars}\times Imbalance$	0.054	
	(0.066)	
$AvgRating$		0.039*
		(0.021)
$Ratin{g}_{1 Star}\times AvgRating$		-0.015
		(0.023)
$Ratin{g}_{2 Stars}\times AvgRating$		-0.010
		(0.020)
$$Ratin{g}_{3 Stars}\times AvgRating$$		(omitted)
$Ratin{g}_{4 Stars}\times AvgRating$		0.017
		(0.017)
$Ratin{g}_{5 Stars}\times AvgRating$		0.013
		(0.020)
$\mathrm{ln}(RevExperience)$	0.166***	0.165***
	(0.001)	(0.001)
$Ratin{g}_{1 Star}$	0.259***	0.259***
	(0.009)	(0.009)
$Ratin{g}_{2 Stars}$	0.202***	0.202***
	(0.008)	(0.008)
$Ratin{g}_{3 Stars}$	(omitted)	(omitted)
$Ratin{g}_{4 Stars}$	-0.212***	-0.213***
	(0.006)	(0.006)
$Ratin{g}_{5 Stars}$	-0.344***	-0.347***
	(0.006)	(0.006)
Constant	6.227***	6.228***
	(0.034)	(0.034)
Pseudo-R-squared	0.014	0.015
Observations	373,205	373,205
Model	ZTNB	ZTNB

Three-star ratings are used as base for the individual rating and the interaction terms and are therefore omitted. Both columns represent a zero-truncated negative binomial regression model (ZTNB). Pseudo-R-squared represents McFadden’s pseudo-R-squared. Robust standard errors clustered at the restaurant level in parentheses. ^***^ p<0.01, ^**^ p<0.05, ^*^ p<*0.1*

In addition, Column (ii) provides the robustness check analogous to the Google Maps data where we use the average rating valence instead of the imbalance score. Although the main effect for $AvgRating$, capturing the interaction with three-star ratings, exhibits a statistically significant coefficient ($p=0.06$), none of the other interaction terms is statistically significant. Hence, we consistently find no support for Hypothesis 4.

### Contrasting our findings with Li et al.’s (2020) findings

As outlined in the related literature, Li et al. (2020) also examined the interplay between expectation disconfirmation and textual review length. The authors also relied on a Yelp dataset, and observed that the textual review length was positively associated with negative disconfirmation. Applying the same logic as Li et al. (2020) to the Yelp data used in Sect. ‘Cross-validation with another review dataset’, we were able to replicate the positive and statistically significant effect for Li et al.’s (2020) definition of negative disconfirmation (i.e., all ratings that are below the average rating). With our logic to test the effect of disconfirmation on textual reviewing effort, however, do not observe that experiencing disconfirmation is significantly associated with longer textual reviews. In this context, it is important to note that the identification of disconfirmation differs considerably between Li et al. (2020) and this study. We focused on the interaction between reviewers’ own experiences and the rating valence of existing reviews. Li et al. (2020), in contrast, assigned all reviews that deviate from the average rating (rounded to the nearest half-star) as “disconfirming.” Hence, they define all reviews with ratings 0.5 stars lower (higher) than the average rating as negative (positive) disconfirmation. For instance, for a location with an average rating of four stars, their approach would assign all one-star, two-star, and three-star ratings as reviews with negative disconfirmation. All five-star ratings would represent reviews with positive disconfirmation. Hence, this definition of disconfirmation is substantially broader than our definition and does, in our opinion, not necessarily capture only disconfirmation. It can be the case that Li et al.’s (2020) findings regarding disconfirmation might also be driven by the observation that low-valence reviews are typically longer than high-valence reviews (e.g., Chua & Banerjee, 2015; Ghasemaghaei et al., 2018 or Salehan & Kim, 2016). Notably, we can also observe this pattern for both the Google Maps dataset and the Yelp dataset with the main effects of the one-star ratings on textual review length being statistically significant and positive. Consequently, it is necessary, in our opinion, to examine the combination of the rating valence of existing reviews and reviewers’ own experience for identifying the potential effects of expectation disconfirmation. In other words, textual reviews of one-star ratings do not necessarily have to be the result of a negative disconfirmation. They might simply indicate that reviewers were not satisfied with the service or the product and therefore outline their own bad experiences.

## Discussion and conclusion

This study emphasizes the importance of existing reviews to future textual reviewing effort. First, we observe that the sole number of existing reviews matters. A high number of existing reviews is associated with both a lower propensity to write an optional textual review (i.e., Hypothesis 1) and a shorter length of a potential textual review (i.e., Hypothesis 2). Thus, both hypotheses developed from the collective effort model by Karau and Williams (2001), can be confirmed. If a potential reviewer observes that a high number of reviews already exists, her individual contribution will not have as large of an impact on the collective task of reviewing. Consequently, she will invest less effort in the textual review. In contrast, if the number of existing reviews is low, the individual review will be much more visible and impactful, making the reviewer put more effort into the collective task.

Second, we do not observe that expectation disconfirmation matters for the reviewing effort, i.e., a reviewer does not invest more effort if her own experience is opposed to the rating valence of existing reviews. Hence, we do not find support for Hypotheses 3 and 4. Put another way, the propensity to write a textual review and actual textual review length are independent of a potential expectation disconfirmation. Notably, while we observe that both negative experiences (i.e., one- and two-star ratings) as well as very good experiences (i.e., five-star ratings) increase the propensity to write an optional textual review, only negative experiences also increase the textual review length. We observe this latter pattern for both Google Maps and Yelp data.

To examine the potential reasons why we do not find support for Hypotheses 3 and 4, we also examined the textual content of reviews. More specifically, we compared the content of reviews expected to exhibit strong disconfirmation (e.g., one-star and two-star ratings with high imbalance scores, or five-star and four-star ratings with low imbalance scores) with reviews exhibiting less disconfirmation (e.g., one-star and two-star ratings with low imbalance scores, or five-star and four-star ratings with high imbalance scores). While we sporadically observe references to the opposed rating valence (e.g., “I’m a little confused as to why I’ve seen so many bad reviews on here”), we acknowledge that these reviews are not longer than others, and they do not try to “restore balance” as expected by theory. In fact, reviews with low ratings typically focus on outlining their own bad experiences, independent of the rating valence of the existing reviews.

### Theoretical implications

From a theoretical perspective, our results contribute to the understanding of the determinants of the textual reviewing effort invested by reviewers. First, our findings suggest that the underlying mechanism of reviewers when they observe existing reviews can be described with the collective effort model of Karau and Williams (2001). Thus, reviewing can be seen as a collective task in which we observe the phenomenon of social loafing. Importantly, Dellarocas et al. (2010) also observed a higher propensity to review at all if products were popular (i.e., typically products with a high number of reviews), suggesting a U-shaped relationship between the propensity to review and their measure of product popularity. As we do not observe this pattern for the number of existing reviews independent of the proxy for textual reviewing effort,^4^ we conclude that the collective effort model is applicable to explain the underlying cognitive mechanism.

Second, our results concerning Hypotheses 3 and 4 suggest that the expectation disconfirmation theory is not applicable to explain textual reviewing effort. We do not observe increased textual reviewing effort in the case of high disconfirmation, neither for the two proxies of reviewing effort, nor for either of the two datasets. Although this contradicts Li et al.’s (2020) findings, controlling for individual ratings is, in our opinion, necessary to identify the effect of expectation disconfirmation. An explorative analysis of the textual content of reviews expected to exhibit strong disconfirmation indicates that there are only very few incidences in which reviewers refer to the opposed rating valence. Furthermore, these reviews are not longer, and reviewers focus on their own bad experiences. Hence, it seems that expectation disconfirmation theory is applicable to predict whether a reviewer is likely to submit a positive or negative rating (Ho et al., 2017), but not to explain the textual reviewing effort. Consequently, this observation also adds to the argument by Hennig-Thurau et al. (2004), i.e., that (among other motives) reviewers submit a review to vent negative feelings and bad experiences (i.e., one-star ratings and two-star ratings) exhibit more textual reviewing effort; nevertheless, this is independent of whether a disconfirmation is experienced or not.

### Practical implications

The practical implications of our study are that review system designers need to consider not only consumers (who are observing existing reviews) but also potential reviewers. Thus, while providing detailed information regarding existing reviews is helpful for consumers, it might also make potential reviewers invest less effort. Thus, review system designers face an important trade-off because helpful reviews mainly depend on the reviewing effort invested. To mitigate the effect of social loafing, they might incorporate adjusted design features, such as omitting the total number of existing reviews when showing the average rating or displaying only a few highlighted reviews on the first page. This might increase the reviewers’ feeling that they are making a significant contribution to the collective task of reviewing. With such a feature, potential reviewers may not perceive that their individual reviews have no impact on the collective task. Similarly, review system designers could also segment reviewers into subgroups so that each reviewer only sees the existing reviews from other reviewers of the same subgroup (e.g., based on age, language, or purpose of purchase).

In addition, online platforms with review systems should consider incorporating an expiration date to reviews. In this way, a review system does not have to actively delete reviews from reviewers but still ensures that reviews are up-to-date while avoiding an ever-growing number of reviews. Online platforms could also use review expiration as a reminder to revisit a location and provide a new review for the review system. If old reviews are never deleted, it is likely that many reviews are outdated and no longer helpful and, according to our findings, lead to less effort towards creating future reviews.

Regarding the influence of the rating valence of existing reviews, some review system designers already incorporate a design feature for highlighting the polarity of reviews (e.g., Amazon’s “Top positive review” and “Top critical review”). While this might help potential consumers weigh the pros and cons of the respective product or service, our findings (from a broader perspective) suggest that it does not systematically impact future reviewers’ textual reviewing effort.

We conclude that review system designers must balance providing as much detailed information to consumers as possible with ensuring to attract helpful and high-quality reviews from future reviewers. Therefore, the insights gained from our research can help review system designers to understand this trade-off. This can serve to improve review helpfulness in particular and online reputation management in general. Overall, it shows that both newly founded online platforms (see, e.g., Hesse & Teubner, 2020, for a discussion on reputation transfer) and well-established online platforms face problems with review systems.

### Limitations and future research

Our research has some limitations that offer fruitful possibilities for future research. First, we focus on review objects that have already been reviewed. For sights that have not yet been reviewed, the underlying cognitive mechanism might be different, and could be addressed in future research. Similarly, owing the nature of our data collection method with both datasets, we only focus on the two most salient features of existing reviews (i.e., the number of existing reviews and rating valence of existing reviews), and do not account for contextual factors such as highlighted textual reviews. Future research could explicitly focus on how existing (and highlighted) textual reviews influence reviewing effort.

Second, both datasets only address cases in which a reviewer has already decided to review. If we assume that the initial decision of a reviewer to review also represents some type of reviewing effort, our findings might underestimate the total effect of existing reviews on the reviewing effort. Furthermore, the rating valence of existing reviews is positive for both datasets. This means that the probability of a reviewer experiencing positive disconfirmation is low. For future research, it could be valuable to use a dataset that allows for examining the propensity to review at all, and that exhibits more “balanced” rating valences to more easily test the effects of disconfirmation. Proprietary e-commerce data including consumers’ purchases and reviewing histories would represent an attractive environment for such research.

Finally, our findings suggest that the collective effort model accurately describes the underlying cognitive mechanism that reviewers undergo when reviewing an object and perceiving the number of existing reviews. There is, however, no support for the hypotheses regarding the rating valence of existing reviews. One possible limitation might be the assumption that reviewers develop their expectations from existing reviews. This assumption is, however, not necessarily true for all reviewers as they might also use other information sources or simply not develop any expectations. In this context, future research could examine how strong the expectations developed from the rating valence of existing reviews are and whether other sources develop even stronger expectations. Furthermore, future research should also examine the textual content of reviews whose reviewers have experienced high disconfirmation in more detail. While our preliminary inspection of the textual content provides a first step in this direction, more elaborate text-mining approaches should be applied to investigate whether, e.g., the arguments of reviewers differ. This is particularly worthwhile given the contradictory findings on disconfirmation between this study and Li et al.’s (2020) study.

## Appendix

**Table A1:** List of sights used in main analysis

Bridges		Squares	
Erasmusbridge	Rotterdam (Netherlands)	Place Masséna	Nice (France)
Pont du Pierre	Bordeaux (France)	Piazza Solferino	Turin (Italy)
25 de Abril Bridge	Lisbon (Portugal)	Place de la Bastille	Paris (France)
Old Town Bridge	Trondheim (Norway)	Paradeplatz	Zürich (Switzerland)
Ponte Sisto	Rome (Italy)	George Square	Glasgow (United Kingdom)
Ponte Milvio	Rome (Italy)	Leicester Square	London (United Kingdom)
Elisabeth Bridge	Budapest (Hungary)	Place Bellecour	Lyon (France)
Eiserner Steg	Frankfurt (Germany)	Plaça Sant Jaume	Barcelona (Spain)
Puente de Isabel II	Seville (Spain)	Piazza Barberini	Rome (Italy)
Pont Neuf	Paris (France)	Dam Square	Amsterdam (Netherlands)
Fountains		Gates**/**Monuments	
Fontaine Saint-Michel	Paris (France)	Porta Ticinese	Milan (Italy)
Fountain of Neptune	Madrid (Spain)	Puerta de Toledo	Madrid (Spain)
Fontana dei Quattro Fiumi	Rome (Italy)	Marble Arch	London (United Kingdom)
Neptune Fountain	Berlin (Germany)	Rua Augusta Arch	Lisbon (Portugal)
Diana, Princess of Wales Memorial Fountain	London (United Kingdom)	Arch of the Sergii	Pula (Croatia)
Fountain of Neptune	Bologna (Italy)	Freedom Monument	Latvia (Riga)
Cascada Monumental	Barcelona (Spain)	Monument aux Girondins	Bordeaux (France)
Morosini fountain	Crete (Greece)	Liberty Statue	Budapest (Hungary)
Fonte Luminosa	Lisbon (Portugal)	Chopin Statue	Warsaw (Poland)
Jet d’Eau	Geneva (Switzerland)	Emperor William Monument	Porta Westfalica (Germany)

Sight names are derived from English Wikipedia pages

## Footnotes

1 Burtch et al. (2018) examined the effect of actively providing the number of previously written reviews to reviewers on the reviewing effort. Their setting, however, is very different from our setting, as they directly sent reviewers the information on how many reviews were submitted by other reviewers to deliberately provide a social norm.

2 As our observation period covers an entire year, and one could argue that textual reviewing effort might vary during the year. For instance, reviewers might be less likely to write textual reviews in winter months, as they might be wearing gloves. Similarly, some fountains might be switched off during winter, leading reviewers to complain.

3 Note that we prefer a negative binomial regression model over a zero-inflated model as the latter assumes two different sources of “zeros.” This is, however, not the case for our data: if a reviewer decides to not write a textual review, textual review length is zero. If she decides to write a textual review, on the other hand, textual review length has to be greater than zero. Hence, there is only one source for a textual review length of zero.

4 We explicitly confirmed this regarding the existence of a U-shaped relationship by replacing $\mathrm{ln}(NumReviews)$ with an indicator variable that categorizes the number of existing reviews into three bins (low number of existing reviews, medium number of existing reviews, and high number of existing reviews). We observe (i) a positive and significant coefficient for the indicator variable for the low number of existing reviews, (ii) an insignificant coefficient for the medium number of existing reviews, and (iii) a negative and significant coefficient for the high number of existing reviews for both proxies of textual reviewing effort (not tabulated). Hence, we conclude that the effect of the number of existing reviews does not exhibit a U-shaped pattern.
